# Supplementary material for: In Silico and In Vivo Evaluation of the Maqui Berry (Aristotelia chilensis (Mol.) Stuntz) on Biochemical Parameters and Oxidative Stress Markers in a Metabolic Syndrome Model
Source: Metabolites. 2023 Dec 7;13(12):1189. doi: 10.3390/metabo13121189 (PMC10744843; doi:10.3390/metabo13121189)
Supplement: Supplementary file 1 [file metabolites-13-01189-s001.zip › metabolites-2714330-supplementary.pdf]

Supplementary Figure S1

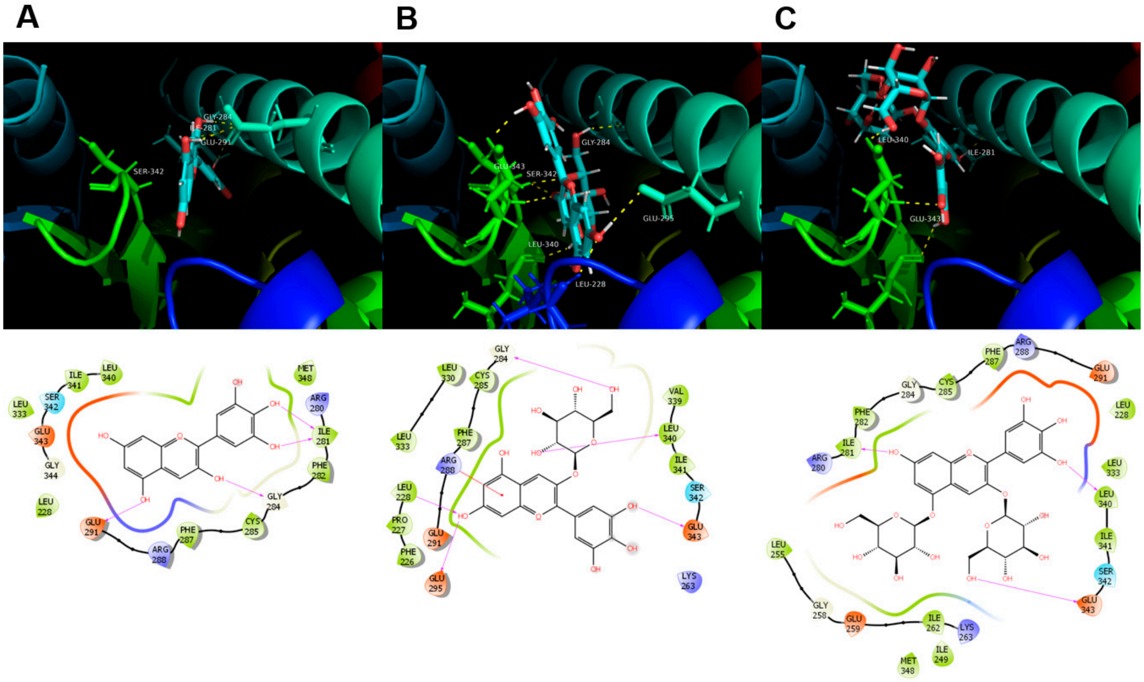

Supplementary Figure S2

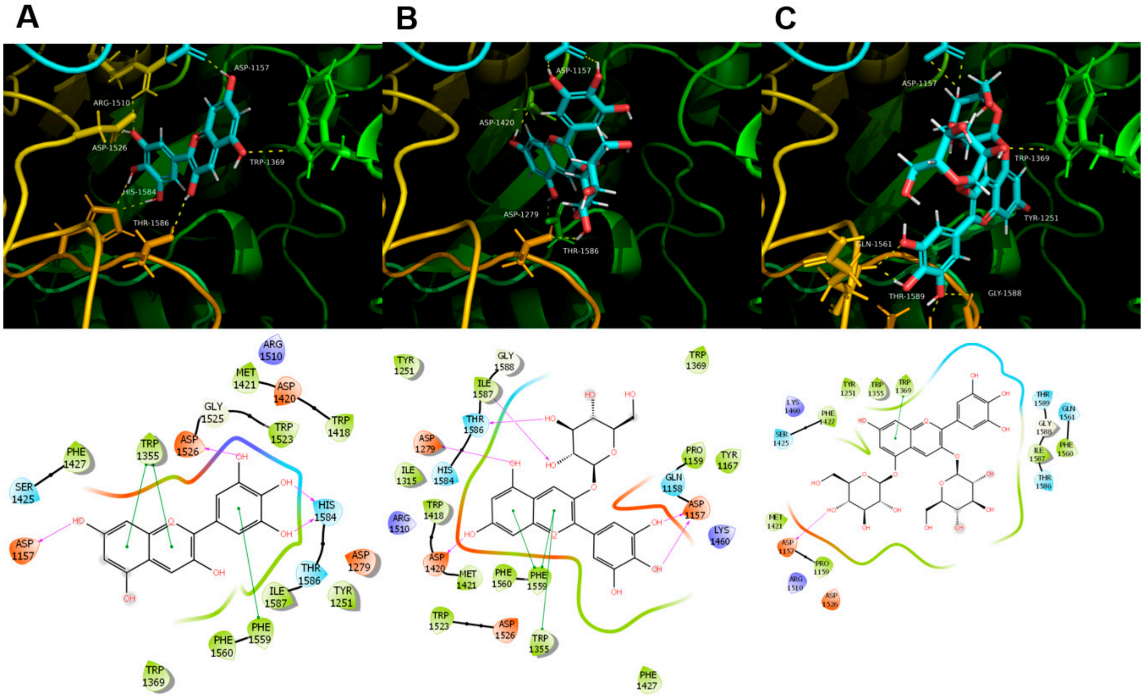

Supplementary Figure S3

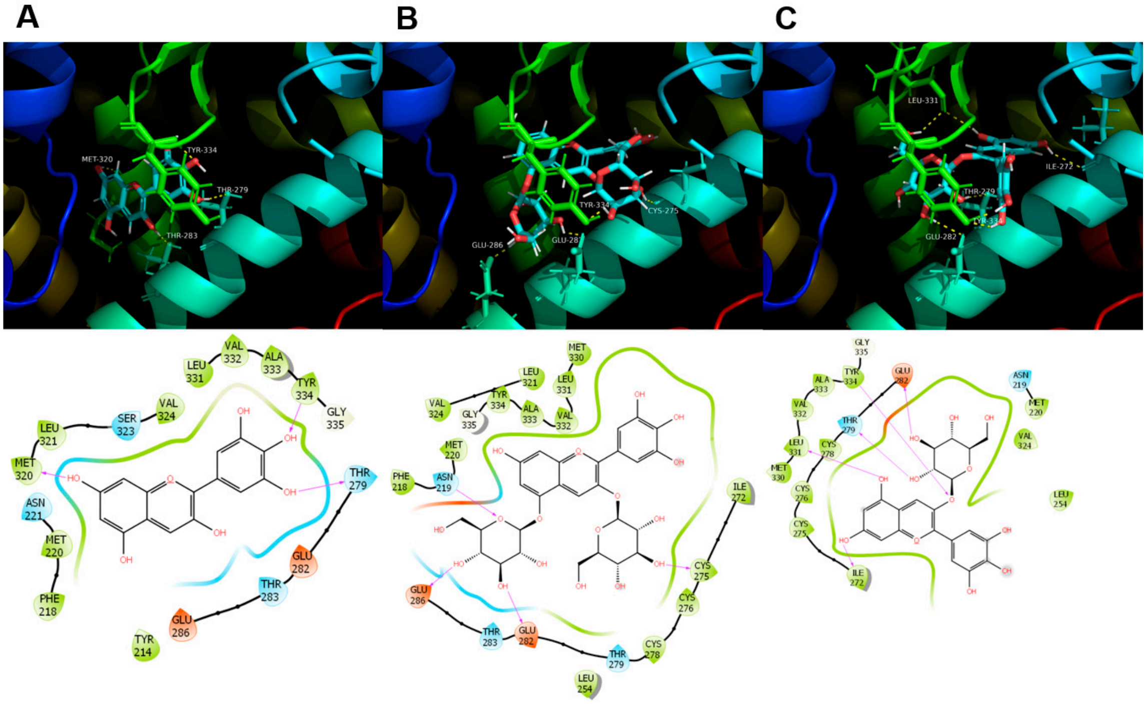

Supplementary Figure S4

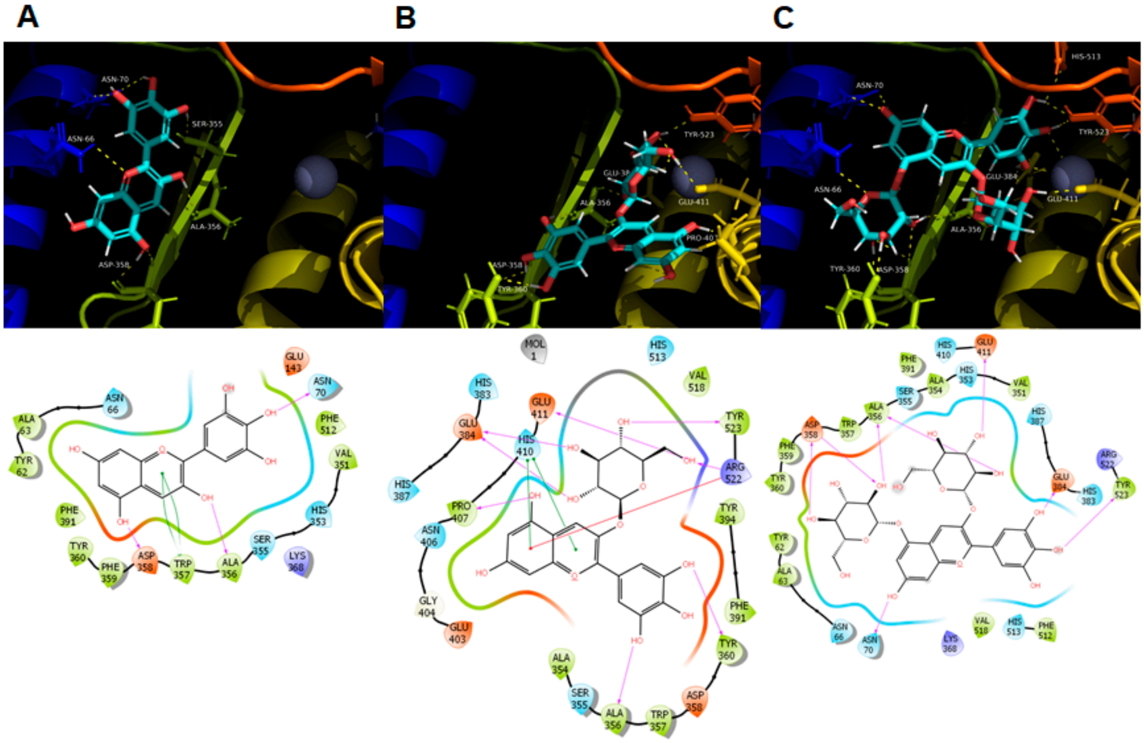

Supplementary Figure S5

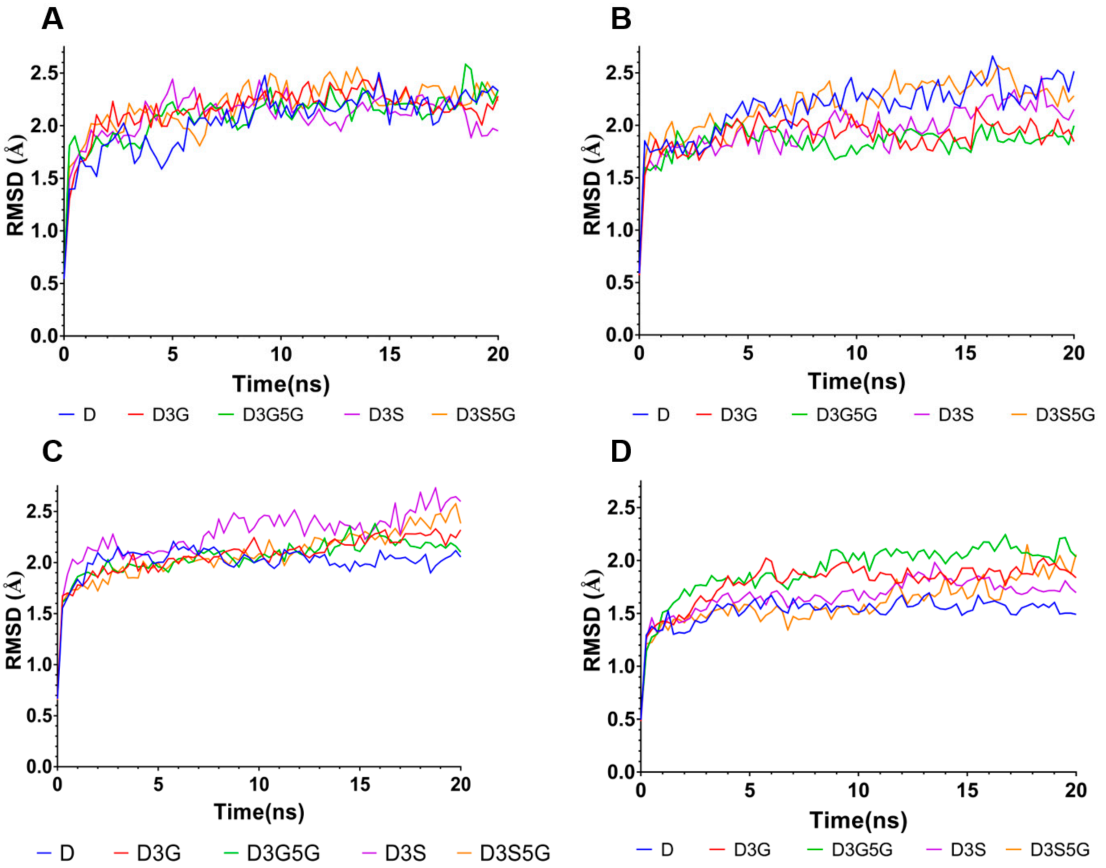

Figure Legends (Supplementary Figures)

Figure S1(SI). Predicted poses of (a) **D**, (b) **D3G**, and (c) **D3G5G** in the active site of the PPAR-g ligand-binding domain. Non-polar amino acids are colored in green, polar residues in cyan, basic in blue, and acid in red. Hydrogen bond interactions are depicted as arrows and π-π or π-cation interactions as dot-ended lines.

Figure S2(SI). Predicted poses of (a) **D**, (b) **D3G**, and (c) **D3G5G** in the active site of the α-glucosidase. See Figure 1-SI for coloring interpretation.

Figure S3(SI). Predicted poses of (a) **D**, (b) **D3G**, and (c) **D3G5G** in the LBD of PPAR-α. See Figure 1-SI for coloring interpretation.

Figure S4(SI). Predicted poses of (a) **D**, (b) **D3G**, and (c) **D3G5G** in the active site of the α-glucosidase. See Figure 1-SI for coloring interpretation.

Figure S5(SI). RMSD variations during MD simulation of delphinidin and its glycosylated derivatives complexes with (a) PPAR-α, (b) PPAR-γ, and (c) α-glucosidase.
